# Living donor liver transplantation for idiopathic portal hypertension with focal nodular hyperplasia

**DOI:** 10.1186/s40792-022-01428-3

**Published:** 2022-04-21

**Authors:** Yoshiaki Tanji, Kenei Furukawa, Yosuke Igarashi, Mitsuru Yanagaki, Koichiro Haruki, Yoshihiro Shirai, Tomohiko Taniai, Takeshi Gocho, Norimitsu Okui, Toru Ikegami

**Affiliations:** Division of Hepatobiliary and Pancreas Surgery, Department of Surgery, The Jikei University School of Medicine, 3-25-8, Nishi-Shinbashi, Minato-ku, Tokyo, 105-8461 Japan

**Keywords:** Living donor liver transplantation, Idiopathic portal hypertension, Focal nodular hyperplasia, Hepatocellular carcinoma

## Abstract

The patient was a 61-year-old woman with a history of diabetes mellitus who had undergone ileocecal resection for ascending colon carcinoma 5 years earlier, followed by a postoperative adjuvant chemotherapy with XELOX (capecitabine + oxaliplatin). During follow-up, the liver gradually atrophied, and radiological imaging showed suspicious findings of 20 × 14 mm hepatocellular carcinoma (HCC) in the right lobe of the liver. The patient also underwent endoscopic variceal ligation for the esophageal varices. She was referred to our hospital for living donor liver transplantation (LDLT) due to decompensated liver cirrhosis with HCC. The patient did not have hepatitis B or C, and history of alcohol, suggesting that her liver cirrhosis was caused by a non-alcoholic steatohepatitis. The Child–Pugh score was 10 points (class C) and the Model for End-Stage Liver Disease (MELD) score was 8 points. The possibility of HCC could not be ruled out, and LDLT was performed. Postoperative pathological examination revealed idiopathic portal hypertension (IPH), and the mass lesion was diagnosed as focal nodular hyperplasia (FNH). The postoperative course was uneventful and the patient was discharged on postoperative day 14. This is the first case of liver transplantation for IPH with FNH.

## Introduction

Idiopathic portal hypertension (IPH) is a rare condition characterized by the development of clinical portal hypertension. Imaging studies show signs of portal hypertension; however, liver hardness and portal pressure values are usually normal or slightly elevated [1]. IPH has a relatively good prognosis if esophageal varices are controlled; however, liver failure due to decreased responsiveness to treatment may occur, which has reportedly resulted in death. Therefore, patients with IPH and end-stage liver failure are eligible for liver transplantation [2–4].

Herein, we report a case of living donor liver transplantation (LDLT) performed in a patient with liver failure who had a mass lesion that could not be ruled out as hepatocellular carcinoma (HCC), and postoperative pathological examination revealed the diagnosis of IPH and focal nodular hyperplasia (FNH).

## Case presentation

**Fig. 1:**
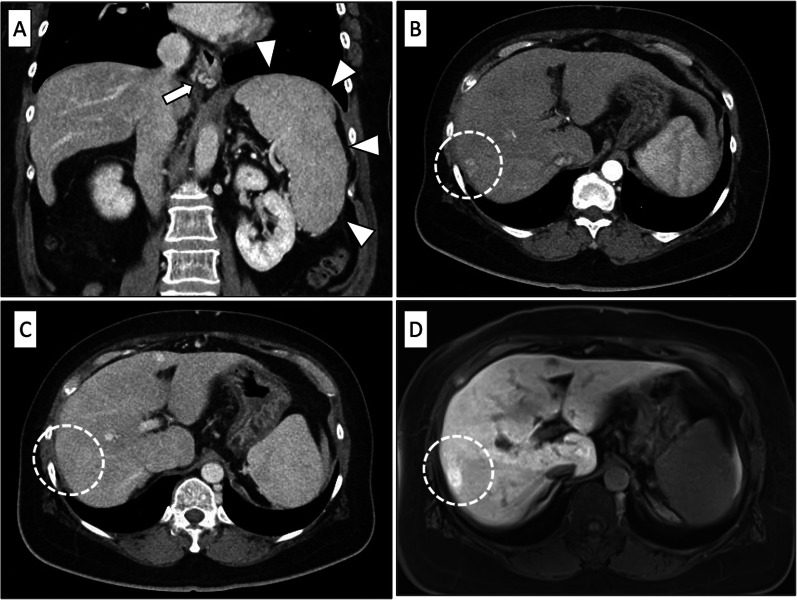
**A** Contrast-enhanced computed tomography showing esophageal varices (arrow) and splenomegaly (arrowheads). **B** Contrast-enhanced computed tomography of early phase showing a mass lesion with early enhancement (circle). **C** Contrast-enhanced computed tomography of portal phase showing a mass lesion without contrast agent wash out (circle). **D** EOB- magnetic resonance imaging of hepatocellular phase showing a mass lesion without contrast agent wash out (circle)

The patient was a 61-year-old woman with a history of diabetes mellitus and hypertension, who had undergone ileocecal resection for ascending colon carcinoma at another hospital 5 years earlier and received XELOX (capecitabine + oxaliplatin, 21 days/course, total 3 courses) and capecitabine (21 days/course, total 5 courses) as adjuvant chemotherapy. She had been under follow-up observation after the surgery; however, the liver gradually atrophied, and a mass lesion of 20 × 14 mm was found in the liver on computed tomography (CT) and Gd-EOB–DTPA magnetic resonance imaging (MRI) examination 1 year before and esophageal varices on esophagogastroduodenoscopy; therefore, endoscopic variceal ligation (EVL) was performed. The mass lesion was followed up, and imaging examination 7 months before showed increased liver atrophy, esophageal varices, splenomegaly, development of collateral blood vessels, and thrombocytopenia. The patient underwent EVL again for esophageal varices and came to our hospital for LDLT because of decompensated liver cirrhosis with portal hypertension and HCC. Contrast-enhanced CT of the abdomen showed mild hepatic atrophy, development of collateral blood vessels, including esophageal varices, splenomegaly, and a small amount of ascites fluid accumulation in the pelvic region (Fig. 1A), and there was a mass lesion with early enhancement at S7/8, and no washout in the portal phase (Fig. 1B, C). Gd-EOB–DTPA MRI also showed a mass lesion with early enhancement in the same area and no washout in the hepatocellular phase (Fig. 1D). Abdominal ultrasonographic findings showed rough surface of liver parenchyma with hepatopetal portal venous flow and an isoechoic mass at S7/8. Laboratory data showed the following: total-bilirubin, 1.7 mg/dL; albumin, 2.4 g/dL; creatinine, 0.58 mg/dL; platelets, 67,000/μL; prothrombin, 50%; prothrombin time-international normalized ratio, 1.5; and non-B and C hepatitis. The Child–Pugh score was 10 points (class C), and the Model for End-Stage Liver Disease (MELD) score was 8 points. Serum tumor markers were within the normal range as follows: carcinoembryonic antigen, 1.7 ng/mL; α-fetoprotein, 3 ng/mL; protein induced by vitamin K absence or antagonist-II, 24 mAU/mL; and carbohydrate antigen 19-9, 16 U/mL. In addition, antinuclear and anti-mitochondrial antibodies were negative.

We diagnosed decompensated liver cirrhosis (Child–Pugh score class C) with HCC. Although liver biopsy was not performed, the patient did not have hepatitis B or C, history of alcohol, and antinuclear or anti-mitochondrial antibodies suggesting that liver cirrhosis was caused by a non-alcoholic steatohepatitis (NASH). Therefore, the patient was judged to be an appropriate candidate for LDLT and surgery was performed. The donor was her son, a 27-year-old man with no pre-existing disease, and a right liver graft was planned for the transplantation. Owing to the positive anti-donor specific antibody, the patient underwent LDLT with preoperative rituximab and mycophenolate mofetil administration, and plasma exchange. The operation time was 489 min, and the intraoperative blood loss was 1030 mL.

**Fig. 2:**
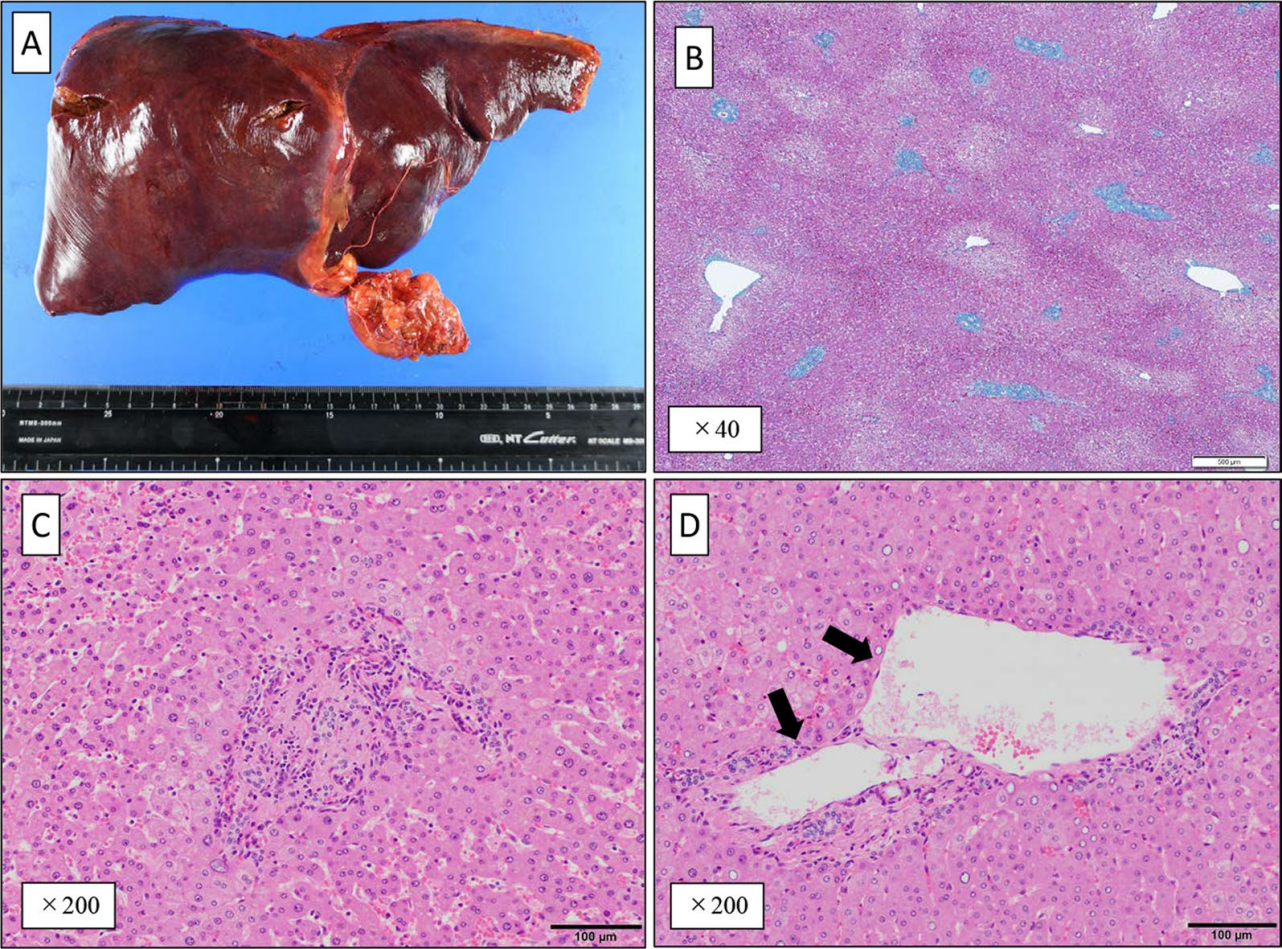
**A** Resected specimen. **B** Pathological diagnosis shows no cirrhosis of the liver, bleeding around hepatic veins, loss of hepatic veins, and no dilation of sinusoids (× 40). **C** Pathological diagnosis shows loss of peripheral portal vein (× 200). **D** Pathological diagnosis shows a new paraportal shunting (arrow) (× 200)

**Fig. 3:**
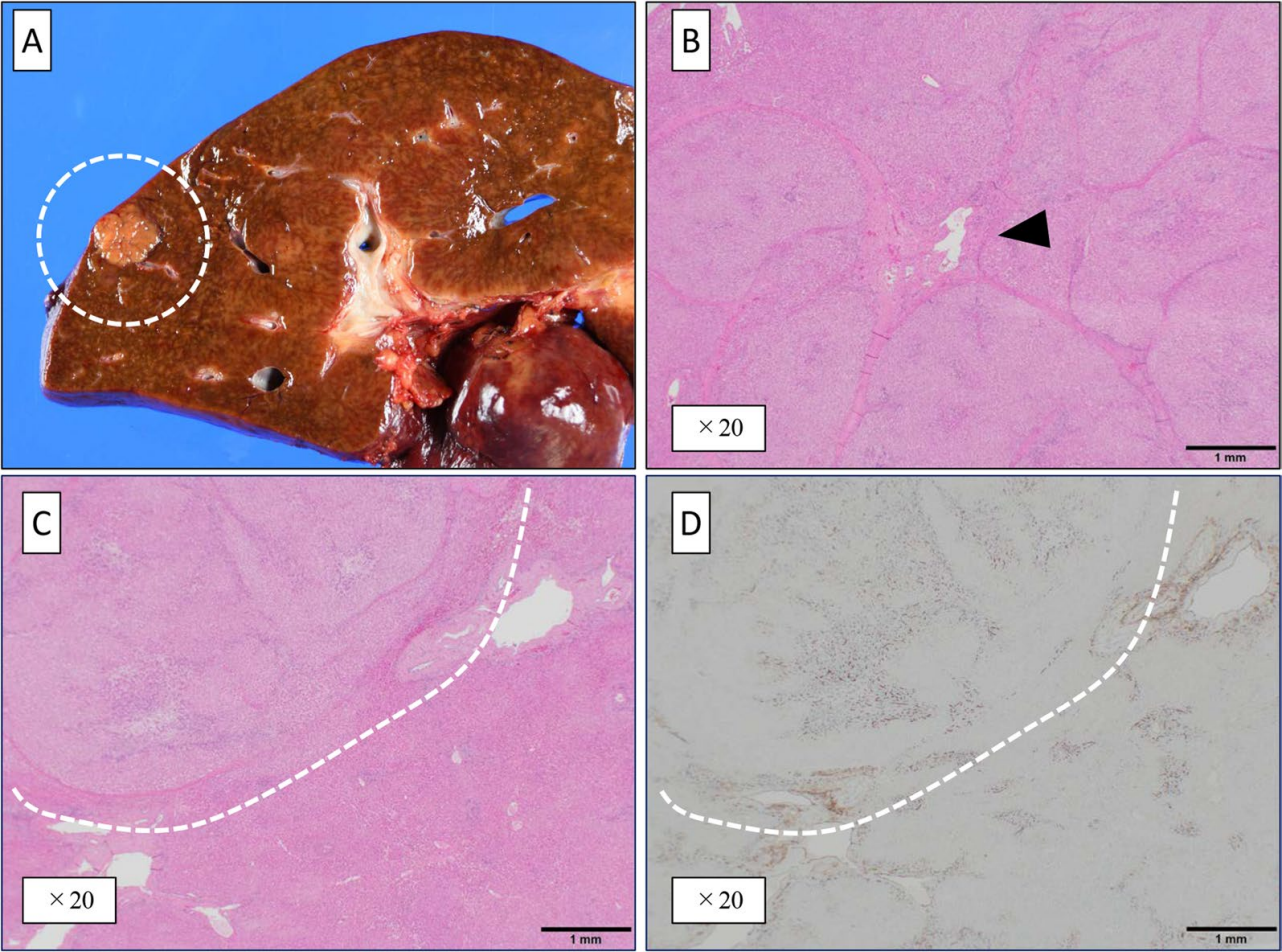
**A** Nodular tumor site on resection specimen (circle). **B** Pathological diagnosis shows nodular lesions, and central scar (arrowhead) (× 20). **C**, **D** CD34 immunostaining showed predominantly arteriogenesis in the nodules (circle) (× 20)

Gross examination of the resected liver revealed no obvious cirrhosis (Fig. 2A). Pathological examination revealed no obvious fibrous septa in the liver parenchyma (Fig. 2B) and no suspicious findings of NASH. In the peripheral portal areas, portal veins were absent (Fig. 2C), and in some peripheral portal areas, new paraportal shunting vessels with thin walls and dilated lumen were observed (Fig. 2D). These were the findings of peripheral portal vein occlusion, and IPH was diagnosed. On the other hand, there were no bleeding around hepatic veins, loss of hepatic veins, and no dilation of sinusoids (Fig. 2B). In addition, a 17 mm diameter nodular lesion on S7/8 (Fig. 3A) was observed on gross examination. Nodular lesions with fibrous septa were observed at the site of the nodular lesions, and a central scar, a characteristic finding of FNH, was also observed (Fig. 3B). CD34 immunostaining predominantly showed arteriogenesis within nodular lesions. Arteriogenesis was also found in non-nodular lesions, in areas with very mild fibrotic changes associated with inflammatory changes. However, more arteriogenesis was observed within the nodules than in the non-nodular lesions (Fig. 3B, D). Based on the above findings, the nodular lesion was diagnosed as an FNH.

The patient recovered satisfactorily with normal liver function and was discharged on postoperative day 14. The patient still has regular follow-up at our hospital with good general condition. The preoperative collateral blood vessels and splenomegaly have disappeared on CT images after LDLT.

## Discussion

IPH is defined as portal hypertension without cirrhosis, and obliterative portal venopathy (OPV) is the primary lesion leading to the development of IPH. Although the cause of IPH is unclear [5], it has been reported to occur in patients with hematologic or autoimmune diseases, human immunodeficiency virus patients on antiviral therapy, colorectal cancer patients on oxaliplatin therapy [1], and inflammatory bowel disease patients on azathioprine therapy [6, 7]. IPH has a relatively good prognosis if esophageal varices are controlled; however, if the patient has end-stage liver failure, liver transplantation is required [2–4]. IPH and OPV are often diagnosed as liver cirrhosis, because they do not have clinical features or characteristic imaging findings; however, they are associated with portal hypertension, such as liver atrophy, splenomegaly, and varicose veins [8]. The fact that the majority of patients with OPV who underwent liver transplantation were also reported to have been preoperatively diagnosed with liver cirrhosis [9] shows the difficulty of preoperative differentiation between IPH or OPV and liver cirrhosis. In the present case, we preoperatively diagnosed decompensated liver cirrhosis with portal hypertension and HCC associated with NASH, and performed LDLT without preoperative liver biopsy.

Considering the timing of deterioration of liver function, oxaliplatin is considered to be the cause of liver dysfunction. There have been reports of sinusoidal obstruction syndrome (SOS) caused by oxaliplatin [10, 11], however, SOS was denied in this case, because pathological findings revealed intact hepatic veins. In addition, the mechanism and pathological changes in IPH caused by oxaliplatin have not yet been elucidated [1].

In our case, we considered the nodular lesion as a possible HCC. However, postoperative pathology revealed that the nodular lesion was an FNH. Localized nodular hyperplasia is a hyperplastic lesion found in the liver without cirrhosis and was first described by Edomondson in 1956 as a localized FNH [12]. In recent years, there has been much debate on how to differentiate between HCC and FNH preoperatively. Even with imaging and preoperative histological diagnosis, it is still difficult to differentiate between the two in some cases [13]. Gd-EOB–DTPA MRI shows high signal in the hepatocellular phase of both green hepatoma and FNH [14]. In addition, it has been reported that approximately 30–60% of FNH cases have no scar in the center of the tumor [15]; therefore, preoperative diagnosis is difficult in some cases. Owing to the absence of central scarring, hepatectomy is performed for diagnostic and therapeutic purposes [16]. In the current case, preoperative Gd-EOB–DTPA MRI showed a mass lesion with early enhancement in the early phase and no washout in the hepatocellular phase; however, there was no obvious central scar on preoperative imaging, making it difficult to preoperatively differentiate HCC from FNH. Postoperative pathological examination revealed a central scar within the nodule, which was consistent with the diagnosis of FNH. Recently, some studies have reported that spectral CT provides a set of quantitative parameters based on monochromatic images, material decomposition images with iodine, and iodine concentration analysis, which may help to improve the accuracy of differentiation between HCC and FNH [17], and it is hoped that further development of preoperative diagnostic techniques will enable us to differentiate between HCC and FNH.

Liver transplantation (LT) for FNH is rare. There have previously been 20 cases including our case (Table 1) [18–32]. Most cases underwent LT due to congenital diseases including congenital absence of the portal vein in children and our case was the first report of LT for FNH complicated with IPH.

**Table 1:** Cases of liver transplantation for focal nodular hyperplasia

Case	Author	Year	Gender	Age	Liver disease	Number of FNH	Maximum size of FNH (mm)
1	I. R. Marino	1992	Female	10	Hepatocellular adenomatosis	Multiple	70
2	K. Tepetes	1995	Female	42	Diffuse FNH	Multiple	ND
3	Fujita S	2006	Female	11	CAPV	Multiple	58
4	Carreiro G	2007	Female	26	Von Gierke disease	ND	ND
5	Okugawa Y	2008	Female	10	BA	1	45
6	Miraglia R	2009	Female	20	Acute liver failure	1	35
7	Merli L	2011	Female	13	Diffuse FNH	Multiple	ND
8	Osorio MJ	2011	Male	7	CAPV	1	130
9	Sanada Y	2015	ND	2	CAPV	2	6
10		2015	ND	3	CAPV	1	80
11		2015	Female	4	CAPV	2	130
12		2015	Male	13	CAPV	6	33
13	Alnajjar A	2015	Male	22	Budd-Chiari	1	120
14	Özden I	2017	Male	17	CAPV	2	80
15		2017	Female	17	CAPV	1	10
16	Xiang W	2019	Female	14	CAPV	Multiple	50
17	Yam MKH	2020	Female	14	CAPV	1	ND
18	Namgoong JM	2021	Female	9	CAPV	Multiple	7
19	Yasunaka T	2022	Female	27	Diffuse FNH	Multiple	ND
20	Our case	2022	Female	61	IPH	1	17

*BA* biliary atresia, *CAPV* congenital absence of the portal vein, *FNH* focal nodular hyperplasia, *IPH* idiopathic portal hypertension

While the cause of FNH has not been fully elucidated, it has been reported that the mechanism of nodule formation is arterial dilatation and increased blood flow due to vascular malformations, which leads to increased nodules [33]. In this case, pathological findings showed the disappearance of the peripheral portal vein and arteriogenesis with CD34 positivity inside the FNH, suggesting that the nodular lesion was formed by a mechanism associated with increased blood flow, in which the occlusion of the peripheral portal vein by IPH led to the formation of a paraportal shunting vessel and regeneration of arteries, which in turn led to the formation of FNH. CD34 immunostaining showed more arteriogenesis within the nodule than in the outside of the nodular lesion, which is consistent with the theory reported in the past that nodular lesions form because of increased blood flow caused by arteriogenesis [34]. This report may help clarify the mechanism of FNH formation in IPH.

## Conclusions

We encountered a case of LDLT for IPH with FNH that was difficult to distinguish from HCC.

## Data Availability

The data presented here are stored in the "Department of Surgery, The Jikei University School of Medicine, 3-25-8, Nishi-Shinbashi, Minato-ku, Tokyo 105-8461, Japan".

## Abbreviations

CT: Computed tomography
EVL: Endoscopic variceal ligation
FNH: Focal nodular hyperplasia
HCC: Hepatocellular carcinoma
IPH: Idiopathic portal hypertension
LDLT: Living donor liver transplantation
LT: Liver transplantation
MELD: Model for end-stage liver disease
MRI: Magnetic resonance imaging
NASH: Nonalcoholic steatohepatitis
OPV: Obliterative portal venopathy
SOS: Sinusoidal obstruction syndrome
